# Changes in Gut Microbiota Associated with Parity in Large White Sows

**DOI:** 10.3390/ani14010112

**Published:** 2023-12-28

**Authors:** Yage Bu, Lingli Feng, Di Xu, Shuai Zhang, Liang Liang, Jinglei Si, Yujie Lu, Qiaoling Liu, Gang Yan, Yubin Wang, Ganqiu Lan, Jing Liang

**Affiliations:** 1 College of Animal Science and Technology, Guangxi University, Nanning 530004, China; ya-gebu@foxmail.com (Y.B.); 15678852652@163.com (L.F.); xudi18788704824@163.com (D.X.); zs714096718@163.com (S.Z.); liangliangjulie@163.com (L.L.); jinglei7139@126.com (J.S.); 15207145840@163.com (Y.L.); 13592622194@163.com (Q.L.); yangang0805@163.com (G.Y.); 18852052688@163.com (Y.W.); gqlan@gxu.edu.cn (G.L.); 2 Guangxi State Farms Yongxin Animal Husbandry Group Co., Ltd., Nanning 530022, China

**Keywords:** litter performance, sows, gut microbiota, parity

## Abstract

**Simple Summary:**

The gut microbiota exhibits distinctive variations among sows at different parities, where parity contributes to 3% of the total dissimilarity among samples (*p* = 0.001). With increasing parity, there is a gradual decline in the relative abundance of bacteria capable of producing short-chain fatty acids (SCFAs), whereas potential pathogenic bacteria become increasingly enriched. The observed alterations in the gut microbiota concerning parity are associated with the aging process in sows.

**Abstract:**

As one of the most critical economic traits, the litter performance of sows is influenced by their parity. Some studies have indicated a connection between the gut microbiota and the litter performance of animals. In this study, we examined litter performance in 1363 records of different parities of Large White sows. We observed a marked decline in TNB (Total Number Born) and NBH (Number of Healthy Born) We observed a marked decline in TNB (Total Number Born) and NBH (Number of Healthy Born) among sows with parity 7 or higher. To gain a deeper understanding of the potential role of gut microbiota in this phenomenon, we conducted 16S rRNA amplicon sequencing of fecal DNA from 263 Large White sows at different parities and compared the changes in their gut microbiota with increasing parity. The results revealed that in comparison to sows with a parity from one to six, sows with a parity of seven or higher exhibited decreased alpha diversity in their gut microbiota. There was an increased proportion of pathogenic bacteria (such as *Enterobacteriaceae*, *Streptococcus*, and *Escherichia–Shigella*) and a reduced proportion of SCFA-producing families (such as *Ruminococcaceae*), indicating signs of inflammatory aging. The decline in sow function may be one of the primary reasons for the reduction in their litter performance.

## 1. Introduction

Sows represent the primary population for commercial pork production, and their performance significantly influences the economic efficiency and competitiveness of breeding facilities [1,2,3]. The parity of sows is a vital factor affecting their reproductive performance in practical production. Higher parities are associated with increased risks of individual mortality, stillborn piglets, the number of piglets born alive, and the number of piglets born weaned compared to lower parities [4,5,6,7].

Recent research in pregnant mice has revealed that maternal gut microbiota can influence the immune and brain functions of their offspring [8]. Disturbances in maternal gut microbiota have been linked to conditions such as Fetal Growth Restriction (FGR), miscarriage, Polycystic Ovary Syndrome (PCOS), endometriosis, and Chronic Pelvic Pain (CPP) [9,10,11,12,13]. Furthermore, a study suggests that *bifidobacterium* impacts maternal body adaptations, placental structure, and nutrient transporter capacity, thereby influencing fetal metabolism and growth [14]. However, whether the changes in the reproductive performance of sows with age (parity) are related to their gut microbes remains unknown.

A recent study highlighted the restructuring of the maternal gut microbiome during pregnancy, which is influenced by parity [15]. However, this study was based on three groups: zero parity, low parity (a parity of one to three), and high parity (a parity of four to seven) groups. Hence, this study did not include comparisons between individual parity groups. In this study, we conducted sequencing analyses of the gut microbiota of 263 Large White sows from different parities, studied the impact of parity on the succession of sow gut microbiota, compared the distinct features of the microbiota across different parities, and screened for microbial markers that may be related to parity and litter performance, providing references for the use and feeding of sows.

## 2. Materials and Methods

### 2.1. Analysis of Litter Performance in Sows of Different Parities

We recorded data on parity, TNB (Total Number Born), and NBH (Number of Healthy Born) for over 1300 Large White sows (including the core selected group and eliminated sows) at a breeding farm in Guangxi. In this study, the Number of Healthy Born refers to piglets born with a weight greater than 1.2 kg. These data were collected along with the respective dates of the events. We employed ANOVA (Analysis of Variance) to test for significant differences in the Total Number Born and Number of Healthy Born offspring among sows of different parities.

### 2.2. Collection of Fecal Samples from Sows of Different Parities

The collection of fecal samples from sows of different parities involved the random selection of 263 sows without recent antibiotic use or vaccination records. We considered the potential impact of pregnancy stage on the gut microbiota. These sows were distributed across distinct pregnancy stages: 60 were in early pregnancy, 60 were in mid-pregnancy, 80 were in late pregnancy, and 63 were in the postpartum stage, ensuring a well-distributed representation across all pregnancy phases. Sampling was conducted intensively over one month, starting at the month’s onset and concluding by its end. The breeding farm’s gestation and farrowing facilities featured fully automated feeding systems, with a single feed line for automated feeding. Throughout the sampling period, the sows were fed a standard commercial diet, devoid of antibiotics or medications, meeting the Nutrient Requirements of Swine standards (GB/T 39235–2020) [16].

The sows were individually housed in a single pen feeding model. Each morning, fresh fecal samples with residual warmth were collected from the floor of individual pens. The central portion of the feces was placed into sampling tubes labeled with the sow’s ID and were immediately stored in a portable icebox. A new pair of PE gloves was used for each sow during the sampling process to prevent contamination. After completion, the fecal samples were promptly frozen at −20 °C. Later, they were transported from the pig farm and stored in liquid nitrogen upon arrival at the laboratory.

### 2.3. Extraction of Fecal Genomic DNA and Sequencing of 16S rRNA

The DNA from fecal samples was extracted using the FastDNA™ SPIN Kit for Soil (MP Biomedicals, Santa Ana, CA, USA). The DNA extraction protocol followed the instructions provided in the kit manual. The DNA quality was assessed through agarose gel electrophoresis, and the V3-V4 region of the 16S rRNA gene was amplified using PCR. The primer sequences for the V3-V4 region were 341F: 5′-CCTACGGGNGGCWGCAG-3′ and 805R: 5′-GACTACHVGGGTATCTAATCC-3′. The target DNA fragments underwent paired-end sequencing using the Illumina Miseq sequencer, with a sequencing strategy of PE250.

### 2.4. Transformed Raw Data into a Feature Table

In this study, the methods described in references [17,18,19,20,21] were employed for the processing and analysis of sequencing data. This involved merging pair-end reads, trimming primers and barcodes, clustering OTUs (Operational Taxonomic Units), de-replication sequences, reducing abundance noise, removing chimeras, and generating the feature table.

### 2.5. Normlization Using the Subsample and the Calculation of Alpha Diversity and Beta Diversity

We employed the methods outlined in [18,22] to calculate alpha diversity and beta diversity. We utilized the vegan package for rarefaction of the feature table. The even depth was set by resampling according to the per-sample prevalence in the feature table and the quantile of sample sequencing depth. The rarefaction depth was set at the minimum sample sequencing depth of 10,000 (95% of the lowest sample sequence count). At this sequencing depth, the observed OTUs encompassed the majority of microbial species within the samples. Further increasing sequencing depth did not result in the inclusion of additional microbial types.

We generated boxplots for various alpha diversity indices and rarefaction curves using R script. We performed ANOVA (Analysis of Variance) tests, followed by post-hoc tests (Tukey–Kramer tests) for multiple comparisons of alpha diversity. We performed CPCoA (Constrained Principal Coordinate Analysis) tests on the Bray–Curtis distance matrix using R script.

### 2.6. Generation of the Relative Abundance Table

In this study, the method outlined in references [18,21] was employed to normalize the feature table and generate the relative abundance table. Normalization of the read counts in the formatted feature table was conducted using the ‘scale’ function in R script [17,20]. The selected OTUs were used to generate a species abundance table for subsequent analyses. A stack plot at the family level was created using R script.

### 2.7. Construction of a Phylogenetic Tree

To gain further insights into the distribution of OTUs (Operational Taxonomic Units) among different parities, OTUs with relative abundances greater than 0.2 were selected. We conducted sequence alignment using the Muscle v5 software for OTUs with an average relative abundance greater than 0.2% and subsequently constructed a maximum likelihood phylogenetic tree using IQ-TREE [18,23].

### 2.8. Random Forest Regression

We constructed a random forest regression model between the gut microbiota and the parity of sows following the methods outlined in reference [24]. Following the approach outlined in reference [24], the gut microbiota data from 263 sows were included in the random forest regression model, which underwent 10-fold cross-validation. Over 100 iterations, the taxa were ranked in order of importance based on the Random Forests algorithm. Upon using 20 significant taxa, the number of taxa reached a point of stability in the cross-validation error curve. Consequently, we selected the top 20 taxa that were significantly associated with parity as marker taxa. These pivotal taxa were presented concerning their contributions using the ggplot2 package (v. 3.4.4). Their distribution across different parities were depicted using a heatmap.

### 2.9. Functional Prediction of the OTU Table Using PICRUSt2

We utilized PICRUSt2 [25] to predict the relative abundance of KEGG metabolic pathways in the gut microbiota. A Spearman correlation analysis was utilized to investigate the relationship between the key taxonomic features of the gut microbiota among different parities and the differential KEGG metabolic pathways of gut microbiota. Multiple group comparisons of metabolic pathways were conducted using ANOVA (Analysis of Variance) and post hoc tests (Games–Howell tests). Benjamini–Hochberg FDR (False Discovery Rate) correction was applied to the *p*-values.

## 3. Results

### 3.1. Variations in Litter Performance across Different Parities of Sows

**Figure 1:**
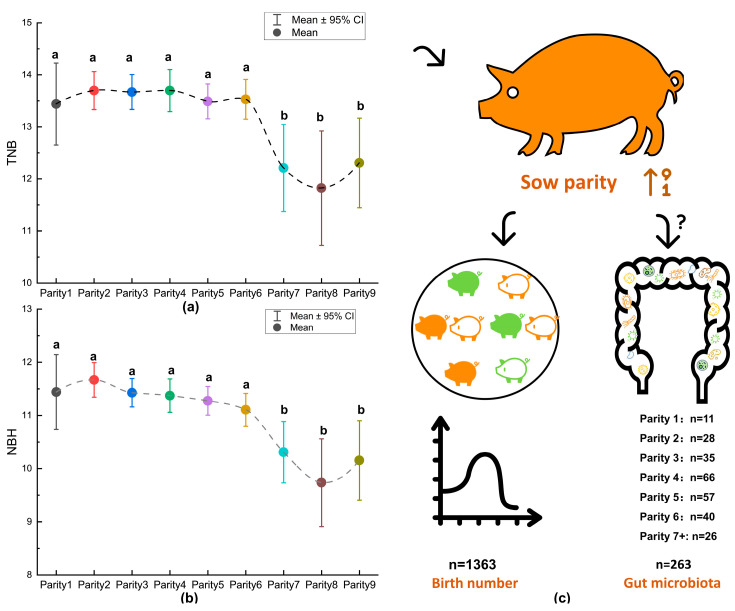
Variation in litter performance among sows of different parities. (**a**,**b**): ANOVA followed by post hoc testing using the Duncan method was employed to conduct multiple comparisons of TNB and NBH among different parity groups. The use of the same letter indicates non-significant differences (*p* > 0.05), whereas different letters indicate significant differences (*p* < 0.05). (**c**): Out of the 1363 records examined, a subset of 263 individuals representing various parities was randomly selected for subsequent analyses of gut microbiota.

In this study, a total of 1363 reproductive records from different parities of Landrace sows in a pig farm in Guangxi were compiled. The results revealed variations in the litter performance among sows of different parities, notably pointing to a significant decrease in the TNB (Total Number Born) and NBH (Number of Healthy Born) in sows with a parity of seven or more (Figure 1a,b).

To gain further insights into the potential changes in gut microbiota across sows of different parities and the association between gut microbiota and litter performance, a subset of 263 sows from the pool of over 1300 individuals, representing different parities, were randomly selected for gut microbiota sequencing and analysis. This subset comprised eleven sows with a parity of one, twenty-eight sows with a parity of two, thirty-five sows with a parity of three, sixty-six sows with a parity of four, fifty-seven sows with a parity of five, forty sows with a parity of six, twelve sows with a parity of seven, seven sows with a parity of eight, and seven sows with a parity of nine (Figure 1c).

### 3.2. Variations in Gut Microbiota among Different Parities

**Figure 2:**
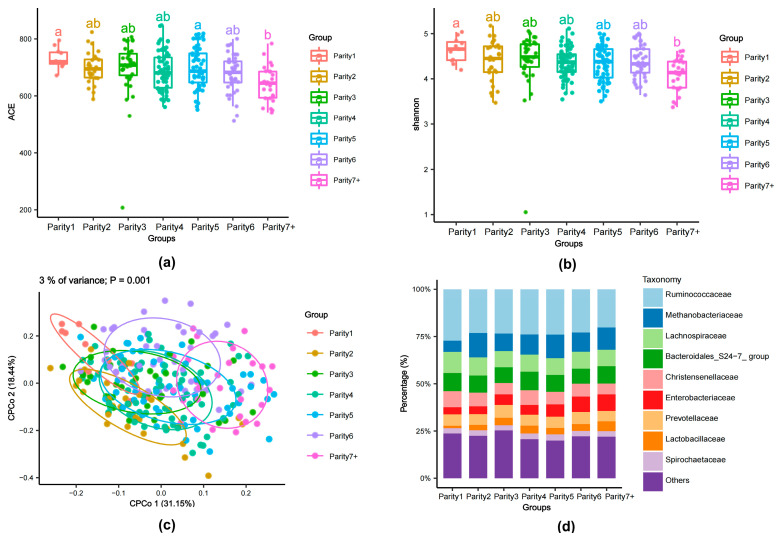
The composition of gut microbiota among sows of different parities. (**a**): Multiple comparisons of ACE were conducted using ANOVA, followed by the Tukey–Kramer post hoc test. The use of the same letter indicates non-significant differences (*p* > 0.05), whereas different letters indicate significant differences (*p* < 0.05). (**b**): Multiple comparisons of Shannon index were conducted using ANOVA, followed by the Tukey–Kramer post hoc test. The use of the same letter indicates non-significant differences (*p* > 0.05), whereas different letters indicate significant differences (*p* < 0.05). (**c**): CPCoA (Constrained Principal Coordinate Analysis) of Bray–Curtis distance matrices. The top 3% of variance represents the displayed plane coordinates, with parity as a condition, explaining 3% of the variation (*p* = 0.001). The *X*-axis label CPCo1 (31.15%) is the first principal coordinate axis, and the *Y*-axis label CPCo2 (18.44%) is the second principal coordinate axis. The percentage of variation indicated in each axis corresponds to the fraction of the total variance explained by the projection. (**d**): The stacked chart illustrates the relative abundance of the top nine taxa at the family level.

The alpha diversity of the gut microbiota among sows of different parities showed subtle trends. There were no significant changes observed in the ACE and Shannon’s diversity index from the group with a parity of one to the group with a parity of six, and no statistical differences were found in the ACE and Shannon’s diversity index among these six groups. In contrast, alpha diversity in the group with a parity of seven or more exhibited a decreasing trend compared to the other six groups, showing significant differences in the ACE and Shannon’s diversity index compared to the group with a parity of one (Figure 2a,b).

To assess the effect of different parities on the sow gut microbiota, we compared the β-diversity using Bray–Curtis distances and conducted a CPCoA (Constrained Principal Coordinate Analysis). The results demonstrate the existence of certain differences in the β-diversity of the gut microbiota among sows of different parities. With the change in parities, the sample variability among different parity groups changed along both the CPCo1 and CPCo2 axes. The gut microbiota of low-parity sows (with a parity of one and a parity of two) and high-parity sows (with a parity of six, seven, or more) exhibited substantial disparities along cPCoA axis 1 and cPCoA axis 2. In contrast, there was less distinction observed among the gut microbiota of sows in the second, third, fourth, and fifth parities along c CPCoA axis 1 and cPCoA axis 2. Parity explained 3% of the total dissimilarity among the samples (*p* = 0.001) (Figure 2c).

The taxonomic stacked bar plot demonstrates that, in comparison to low-parity sows (especially sows with a parity of one), high-parity sows (with a parity of seven or more) have a lower proportion of taxa from families such as *Ruminococcaceae*, *Lachnospiraceae*, *Christensenellaceae*, and *Prevotellaceae*. Conversely, they have a higher proportion of families such as *Enterobacteriaceae* and others (Figure 2d).

### 3.3. Variations in High-Abundance OTUs of Gut Microbiota among Different Parities

**Figure 3:**
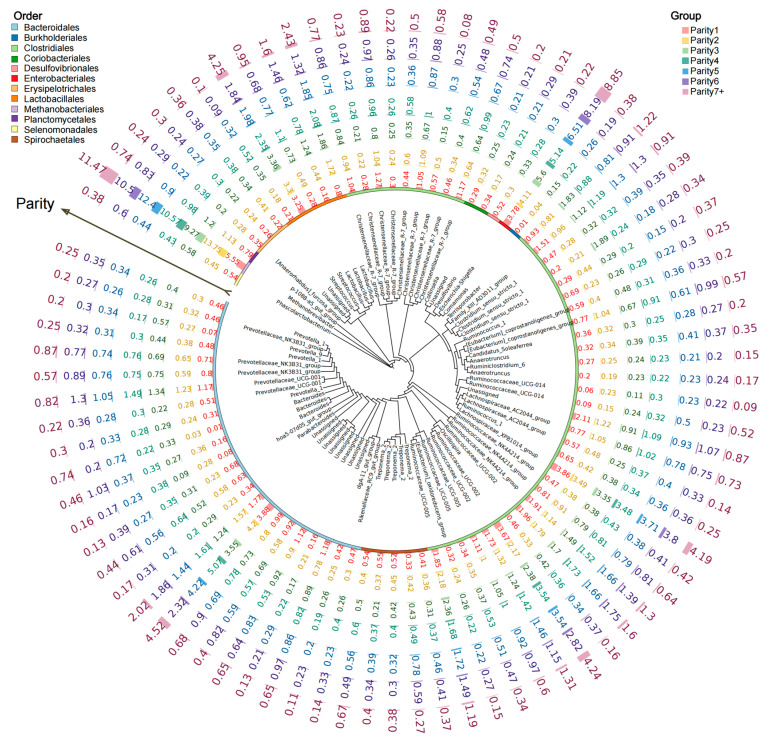
Top OTU members of the bacterial microbiome associated with the different parities. The Maximum Likelihood phylogenetic tree consists of nodes and branches. The terminal nodes of the tree represent the OTUs (Operational Taxonomic Units). To facilitate a clearer understanding of the variations in the relative abundance of high-abundance OTUs among sows of different parities, we annotated and replaced the high-abundance OTUs (IDs) with corresponding Genus-level IDs. Outward from the nodes, the tree demonstrates the order-level classification of these high-abundance OTUs. Further outward, the tree sequentially presents the relative abundance information of the top OTUs from first parity to seven or more parities.

Methanobrevibacter, Streptococcus, Lactobacillus, Escherichia–Shigella, Comamonas, and Ruminococcaceae_UCG-002 exhibited relatively high relative abundances in each parity. With an increase in parity, these genera, including Methanobrevibacter, Streptococcus, Lactobacillus, Escherichia–Shigella, Comamonas, and Ruminococcaceae_UCG-002, also experienced changes in their relative abundances. Notably, Streptococcus exhibited a close phylogenetic relationship with Lactobacillus. Escherichia–Shigella exhibited a similar relationship with Comamonas (Figure 3).

### 3.4. Important Taxonomic Features of Gut Microbiota among Different Parities

**Figure 4:**
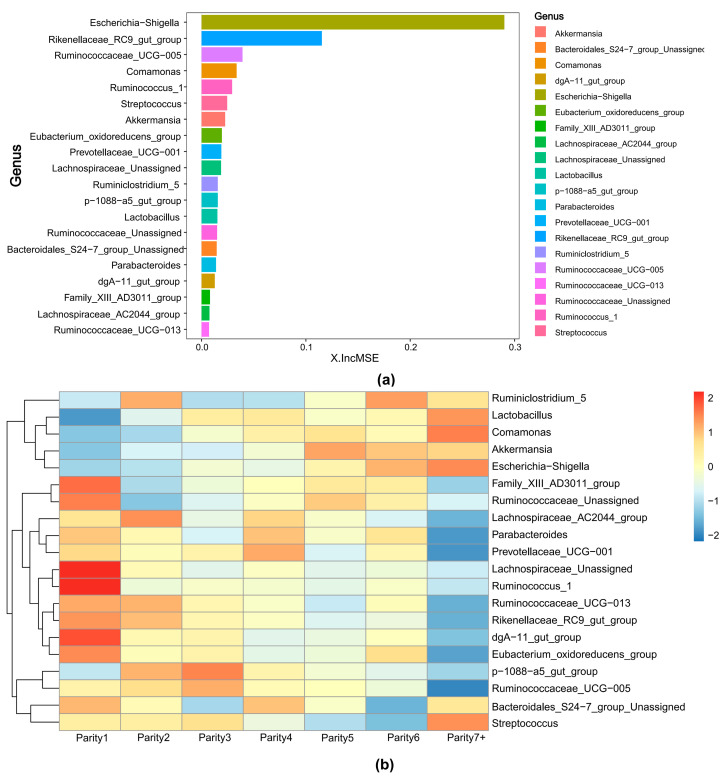
The important taxonomic features that distinguish different parity groups. (**a**): A Random Forest regression model was constructed using the Random Forest package (v 4.7-1.1), and 20 important taxonomic features were selected. (**b**): A heatmap illustrating the trend in the average relative abundance of these 20 features with different sow parities.

Using the random forest regression model, 20 important taxonomic features related to sow parity were identified. These features, ranked by their importance from high to low, included *Escherichia–Shigella*, *Rikenellaceae_RC9_gut_group*, *Ruminococcaceae_UCG-005*, *Comamonas*, *Ruminococcus_1*, *Streptococcus*, *Akkermansia*, *Eubacterium_oxidoreducens_group*, and *Prevotellaceae_UCG-001*, among others (Figure 4a).

*Escherichia–Shigella*, *Akkermansia*, *Lactobacillus*, and *Comamonas* gradually became enriched in the high-parity group. In contrast, the relative abundances of *Ruminococcaceae_UCG-005*, *Eubacterium_oxidoreducens_group*, *Rikenellaceae_RC9_gut_group*, *dgA-11_gut_group*, and *Ruminococcus_1* exhibited a declining trend (Figure 4b).

### 3.5. Analysis of Differential Metabolic Pathways among Gut Microbiota of Different Parities

**Figure 5:**
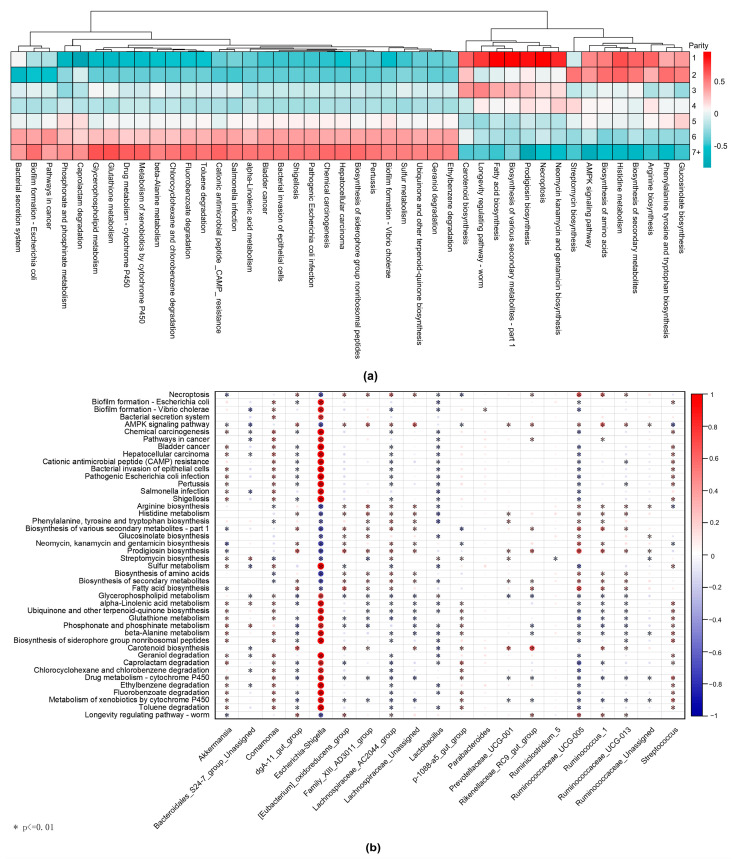
Differential metabolic pathways of gut microbiota among different parities. (**a**): The heatmap illustrates the relative abundance of differential metabolic pathways across different parities. In ascending order from sows with a parity of one to sows with a parity of seven or more, the relative abundance variations of distinct metabolic pathways across different parity groups are displayed from top to bottom. (**b**): The correlation analysis using the Spearman coefficient assessed the associations between the important taxonomic features and the differential metabolic pathways. Asterisks (*) denote significant correlation (*p* < 0.01), where red indicates a positive correlation, blue indicates a negative correlation, and the circle size represents the magnitude of the correlation coefficient.

To gain a better understanding of the functional characteristics of the gut microbiota among different parities, we utilized PICRUSt2 to predict the KEGG pathways of the gut microbiota across different parities. In total, we predicted 311 third-level KEGG metabolic pathways. Among the seven groups, there were 44 differential metabolic pathways (*p* < 0.05, FDR < 0.05) (Figure 5a).

With increasing parity, the relative abundance of the metabolic pathways related to biosynthesis gradually decreased, encompassing pathways such as glucosinolate biosynthesis, phenylalanine tyrosine and tryptophan biosynthesis, arginine biosynthesis, the biosynthesis of secondary metabolites, and carotenoid biosynthesis (Figure 5a).

Conversely, the inflammation-related metabolic pathways gradually became more prevalent in the high-parity group, encompassing pathogenic *Escherichia coli* infection, shigellosis, biofilm formation (by Vibrio cholerae), the bacterial invasion of epithelial cells, and biofilm formation (by *Escherichia coli*) (Figure 5a).

The correlation analysis using the Spearman coefficient assessed the associations between the important taxonomic features and the differential metabolic pathways. The results demonstrate that the top-ranked feature, *Escherichia–Shigella*, in the random forest analysis exhibits a highly significant association with 43 differential metabolic pathways (Figure 5b). *Escherichia–Shigella* showed a highly positive correlation with inflammation-related metabolic pathways, including biofilm formation (by *Escherichia coli*), shigellosis, and pathogenic *Escherichia coli* infection, among others (Figure 5b).

## 4. Discussion

This study used a Large White pig population and investigated changes in the gut microbiota as sows progressed from their first to seventh (or more) parity. The results revealed that the gut microbiota underwent significant changes, starting from the first parity, with more pronounced differences observed between first-parity sows and high-parity sows (sows with a parity of seven or more). In contrast, changes in the gut microbiota of sows with intermediate parities were less evident. The gut microbiota of high-parity sows (sows with a parity of seven or more) demonstrates a noticeable decreasing trend in alpha diversity. Notable taxonomic features that contributed to the differences in gut microbiota among sows of different parities included *Escherichia–Shigella*, *Rikenellaceae_RC9_gut_group*, *Ruminococcaceae_UCG-005*, *Comamonas*, and *Ruminococcus_1*, among others.

Compared with parturient sows, the differences in the gut microbiome of first-parity sows may be related to energy metabolism. A study showed a higher relative abundance of *Methanobrevibacter* and a lower relative abundance of *Rikenellaceae_RC9_gut_group* and *dgA-11_gut_group* in the gut microbiota of multiparous cows compared to primiparous cows [26]. These alterations in the most representative taxa might be associated with the host’s energy metabolism [26]. The changes observed in the gut microbiota of primiparous sows might be associated with the challenge they encounter in distributing nutrients to support maternal growth, in addition to fulfilling the needs of fetal growth and lactation [27,28].

Aging of the body is often accompanied with a decline in the diversity of gut microbes. Recent research has highlighted that a key characteristic of the gut microbiota in aging hosts is a loss of microbial diversity, manifesting as an increase in potential pathogenic bacteria, such as *Enterobacteriaceae* [29,30]. The overgrowth of *Escherichia coli*, a member of *Enterobacteriaceae*, in the gut is a crucial factor accelerating host aging [29]. Administering *Escherichia coli* from mouse feces orally to wild mice results in a significant increase in the expression levels of aging markers in the organs of the wild mice [29]. In this study, *Escherichia–Shigella* shows a highly significant positive correlation with biofilm formation (by *Escherichia coli*), with both their relative abundances gradually increasing in high-parity sows. The enterotype characterized by the predominance of *Escherichia–Shigella* dominates in the gut of elderly individuals and 90-year-olds [31]. Aging is recognized as a significant factor in the continual reorganization of the gut microbiome throughout life [30]. In this context, a consistent and marked divergence of the gut microbial profile from healthy-like states is defined as dysbiosis, primarily indicated by reduced intraindividual diversity (such as alpha diversity), decreased health-associated taxa, and an overabundance of pathobionts [29,30,31,32,33]. Sows with a parity of seven and above often face aging-related issues. In this study, the reduction in the alpha diversity index in the gut microbiota of high-parity sows, along with the enrichment of aging biomarkers (such as *Escherichia–Shigella* [31]), further confirms the correlation between alterations in the gut microbiota of high-parity sows and the aging process in these animals. 

In this study, we found *Escherichia–Shigella*, *Comamonas*, *Akkermansia*, *Streptococcus*, and others are significantly positively correlated with inflammation-related metabolic pathways, such as pathogenic *Escherichia coli* infection, shigellosis, biofilm formation (by Vibrio cholerae), the bacterial invasion of epithelial cells, and biofilm formation (by *Escherichia coli*). Recent research has highlighted that an overgrowth of *Escherichia coli* can lead to a significant increase in the expression levels of aging markers in host tissues and organs [29]. By reducing the relative abundance of pathogenic bacteria, such as *Enterobacteriaceae*, and increasing the diversity of the gut microbiota, the inflammation and aging levels of multiple host tissues and organs can be improved [29]. Microbes associated with inflammation, such as *Escherichia–Shigella*, can alter the intestinal mucus layer through various mechanisms, including their ability to degrade mucins, potentially leading to intestinal inflammation [34,35,36]. Recent studies propose that *Akkermansia* is linked to intestinal inflammation, potentially exacerbating gut inflammation in different immune environments [37,38]. This association is attributed to *Akkermansia’s* ability to degrade mucin, which increases pathogen exposure in the host and promotes microbial infiltration into the intestinal mucosa, thereby stimulating bacteria-induced inflammation [37,38].

Chronic inflammation and dysbiosis in the gut microbiome are hallmarks of aging in the body. Inflammatory markers associated with IBD, such as C-reactive protein (CRP) and circulating cytokine levels, tend to increase with age [39,40,41]. Ghosh further highlights that changes in the gut microbiota associated with age-related health deterioration are linked to inflammation within the body [32]. This shift toward an inflammatory gut environment is more conducive to the competition and enrichment of pathogenic bacteria [32]. The observed imbalance in gut microbiota, potential increases in pathogenic bacteria, and the reduction in bacteria that produce SCFAs (short-chain fatty acids) in high-parity sows (with a parity of seven or more) all suggest signs of inflammatory aging and weakness. Age-related weakening in high-parity sows may be a primary cause of their decreased reproductive function.

Probiotics, such as *Bifidobacterium* and *Lacticaseibacillus*, can delay aging by regulating the body’s immune system [42,43]. A recent study indicated that transferring gut microbiota from young mice to their aged counterparts resulted in specific enrichments, including microbiota involved in producing vitamins B7 and B9, along with an increased presence of the Bifidobacterium shunt pathway [44]. These changes potentially led to a reversal of aging-related features in the gut, eyes, and brain of the aged mice [44]. In this study, we found that the microbial population capable of producing short-chain fatty acids (*Ruminococcaceae*) was enriched in low-parity sows compared to high-parity sows. Further research is needed to explore whether probiotics can be used to delay aging and extend the lifespan of sows.

In this study, we utilized nearly equal fecal samples from sows at various gestational periods within each parity to mitigate the potential impact of gestation duration on the study outcomes. However, due to the limited number of individuals per parity at different gestational time points, further investigation is warranted to comprehensively evaluate the effects of diverse gestational periods on the composition of the gut microbiota.

## 5. Conclusions

Starting from the first parity, the gut microbiota of sows at different parities underwent certain changes. These changes were more pronounced among first-parity sows and high-parity sows (sows with a parity of seven and above), whereas the variations in gut microbiota among sows of intermediate parities were less evident. The enrichment of potential pathogenic bacteria and the reduction of bacteria capable of producing short-chain fatty acids (SCFAs) in high-parity groups are the primary factors contributing to imbalances in the gut microbiota of high-parity sows. These signs suggest that sows with a parity of seven and above may exhibit signs of aging frailty. This may be an important factor contributing to the decline in litter performance observed in high-parity sows. Considering only the factors associated with gut microbiota, it is advisable to use sows with the sixth parity or lower for reproductive production.

## Data Availability

The datasets used and/or analyzed during the current study are available from the corresponding author on reasonable request. The data are not publicly available due to [some of the data being used for another important study].
